# Postoperative Neutrophil-to-Lymphocyte Ratio Change Predicts Survival of Patients with Small Hepatocellular Carcinoma Undergoing Radiofrequency Ablation

**DOI:** 10.1371/journal.pone.0058184

**Published:** 2013-03-14

**Authors:** Jiaqiang Dan, Yaojun Zhang, Zhenwei Peng, Junting Huang, Hengjun Gao, Li Xu, Minshan Chen

**Affiliations:** 1 Department of Hepatobiliary Surgery, Sun Yat-sen University Cancer Center, Guangzhou, People’s Republic of China; 2 State Key Laboratory of Oncology in South China, Sun Yat-sen University Cancer Center, Guangzhou, People’s Republic of China; 3 Department of Oncology, The First Affiliated Hospital, Sun Yat-sen University, Guangzhou, People’s Republic of China; University of Modena & Reggio Emilia, Italy

## Abstract

**Background:**

An elevated preoperative neutrophil-to-lymphocyte ratio (NLR) has been reported to be a prognostic factor for hepatocellular carcinoma (HCC) patients after treatment. However, the clinical implication of postoperative NLR change remains unclear.

**Materials and Methods:**

From May 2005 to Aug 2008, a cohort of consecutive 178 small HCC patients treated with radiofrequency ablation (RFA) was retrospectively reviewed. The NLR was recorded within 3 days before and 1 month after RFA. Baseline characteristics, overall survival (OS) and recurrence free survival (RFS) were compared according to preoperative NLR and/or postoperative NLR change. Prognostic factors were assessed by multivariate analysis.

**Results:**

Compared with preoperative NLR level, postoperative NLR decreased in 87 patients and increased in 91 patients after RFA. No significant differences were identified between two groups in commonly used clinic-pathologic features. The 1, 3, 5 years OS was 98.8%, 78.6%, 67.1% for NLR decreased group, and 92.2%, 55.5%, 35.4% for NLR increased group respectively (*P*<0.001); the corresponding RFS was 94.2%, 65.2%, 33.8% and 81.7%, 46.1%, 12.4% respectively (*P*<0.001). In subgroup analysis, the survival of patients with lower or higher preoperative NLR can be distinguished more accurate by postoperative NLR change. Multivariate analysis showed that postoperative NLR change, but not preoperative NLR, was an independent prognostic factor for both OS (*P*<0.001, HR = 2.39, 95%CI 1.53–3.72) and RFS (*P* = 0.003, HR = 1.69, 95%CI 1.87–8.24).

**Conclusion:**

The postoperative NLR change was an independent prognostic factor for small HCC patient undergoing RFA, and patients with decreased NLR indicated better survival than those with increased NLR.

## Introduction

Hepatocellular carcinoma (HCC), a highly prevalent and lethal cancer, is the sixth most common cancer and the third leading cause of cancer-related death worldwide [1]. Radiofrequency ablation (RFA) has emerged as a new treatment modality and has become a main modality of locoregional therapy because of its effectiveness and safety for small HCC (<5.0 cm), with a 3-year survival rate of 62–77% [2], [3], a low treatment complication rate of 8–9%, and a low treatment mortality rate of 0–0.5% [4], [5], [6]. Extensive clinical researches have indicated that RFA is an effective treatment for small HCC and has an outcome equal to that of surgical resection, but has the advantage in being less invasive over surgical resection [6], [7], [8], [9]. Prognostic factors affecting the survival after RFA mainly included tumor size [10], [11], [12], [13], tumor number [10], [14], safety margin [10], liver function reserved [10], [12], [14], initial tumor response [15], and so on.

Recently, there are increasing evidences that the presence of systemic inflammation correlates with poorer cancer-specific survival in certain cancers. Various markers of systemic inflammatory response, including cytokines, C-reactive protein (CRP), and absolute blood neutrophil or lymphocyte count as well as their ratio such as neutrophil-to-lymphocyte ratio (NLR) have been investigated for their prognostic roles in certain cancer populations [16], [17], [18], [19], [20]. Studies had demonstrated that an elevated NLR may correlate with a poor prognosis in patients with HCC who underwent transcatheter arterial chemoembolization (TACE) [21], curative resection [22] and orthotopic liver transplantation (OLT) [23], [24]. However, these studies only focused on preoperative NLR, and the clinical significance of postoperative NLR change, which may dynamic reflect the change of balance between host inflammatory response and immune response after treatment, is largely unclear.

This study was designed to evaluate whether postoperative NLR change would be a useful predictor for survival in patients with small HCC undergoing RFA.

## Results

### Patients

From May 2005 to Aug 2008, 364 patients with small liver tumor underwent RFA in Department of Hepatobiliary Surgery, Sun Yat-Sen University Cancer Center. Based on our inclusion criteria, 186 patients were excluded from this study. Among these, 66 patients were recurrence after hepatic resection, 48 patients received TACE before RFA, and 35 patients were liver metastasis. In addition, 20 patients with incomplete clinical or laboratory data and 17 patients lost to follow-up within 3 months after RFA were excluded as well. Finally, 178 patients with 210 tumors, who met all of our inclusion criteria, were enrolled in this study. There were 159 males (89.3%) and 19 females (10.7%), with median age of 57 (range 29–82) years old. 150 patients (84.3%) had one nodule, 24 patients (13.5%) had two nodules and 4 patients (2.2%) had three nodules.

The mean of preoperative NLR was 1.9; there were 68 (38.2%) patients with NLR≥1.9 and 110 (61.8%) patients with NLR<1.9. One month after RFA, comparing with the NLR before treatment, the NLR was decreased in 87 (48.9%) patients and increased in 91 (51.1%) patients. The clinic-pathological characteristics of patients with decreased and increased NLR were detailed in Table 1. There were not significant differences between two groups in commonly used demographic and clinic-pathologic characteristics.

**Table 1:** Comparison of clinic-pathologic characteristics of 178 patients according to postoperative NLR change.

Characteristics	NLR	NLR	*P-*value
	increased(n = 91)	decreased(n = 87)	
Age(year)	56.90±12.57	55.38±11.82	0.407
Sex(F/M)	11/80	8/79	0.532
WBC (109/L)	5.12±1.48	5.71±2.06	0.065
PLT (10^9^/L)	130.91±64.71	139.90±73.28	0.387
ALT (u/L)	39.82±15.13	39.41±16.44	0.864
AST (u/L)	43.84±16.15	41.66±15.65	0.362
ALB(g/L)	39.54±4.50	39.82±4.9	0.605
T-bil(umol/L)	21.17±13.90	19.12±9.45	0.255
ALP(u/L)	94.60±54.39	90.34±31.37	0.526
γ-GGT(u/L)	65.13±32.68	60.37±36.41	0.361
PT(s)	13.93±1.74	13.98±1.50	0.835
Tumor number	1.20±0.50	1.16±0.40	0.577
Tumor size(cm)			0.257
<3	51	56	
3–5	40	31	
AFP (ng/mL)			0.411
≥400	19	14	
<400	72	73	
HBeAg			0.188
Positive	78	80	
Negative	13	7	
Baseline NLR			0.245
≥1.9	31	37	
<1.9	60	50	
Child-Pugh class			0.411
A	72	73	
B	19	14	
Safety margin(cm)			0.133
≥0.5	46	54	
<0.5	45	33	

AFP, a-fetoprotein; ALB, serum albumin; ALT, alanine aminotransferase; ALP, alkaline phosphatase; AST, aspartate aminotransferase; γ-GGT, gamma glutamyl transpeptidase; HBeAg, hepatitis B e-antigen; NLR, neutrophil- lymphocyte ratio; PLT, platelets; PT, prothrombin time; RFA, radiofrequency ablation; T-bil, total bilirubin; WBC, white blood cell.

### RFA Complete Ablation and Complications

Complete ablation was depicted at the spiral CT 4–6 weeks after RFA in 169 patients (94.9%, 169/178). For 9 patients with residual viable tumor, 4 patients in NLR decreased group and 5 in NLR increased group (4/87 vs 5/91, Chi square test, *P* = 1.00), a second RFA was given. After the second treatment, they were all achieved complete ablation.

There were no RFA-related mortalities or major complications. Pain and fever were the most commonly complications. 19 patients suffered from moderate/severe pain in NLR decreased group compared with 16 in NLR increased group (19/87 vs 16/91, Chi square test, *P* = 0.475), and 8 patients experienced fever in NLR decreased group compared with 15 in NLR increased group (8/87 vs 15/91, Chi square test, *P = *0.147).

### Impacts of NLR on Overall Survival

**Figure 1:**
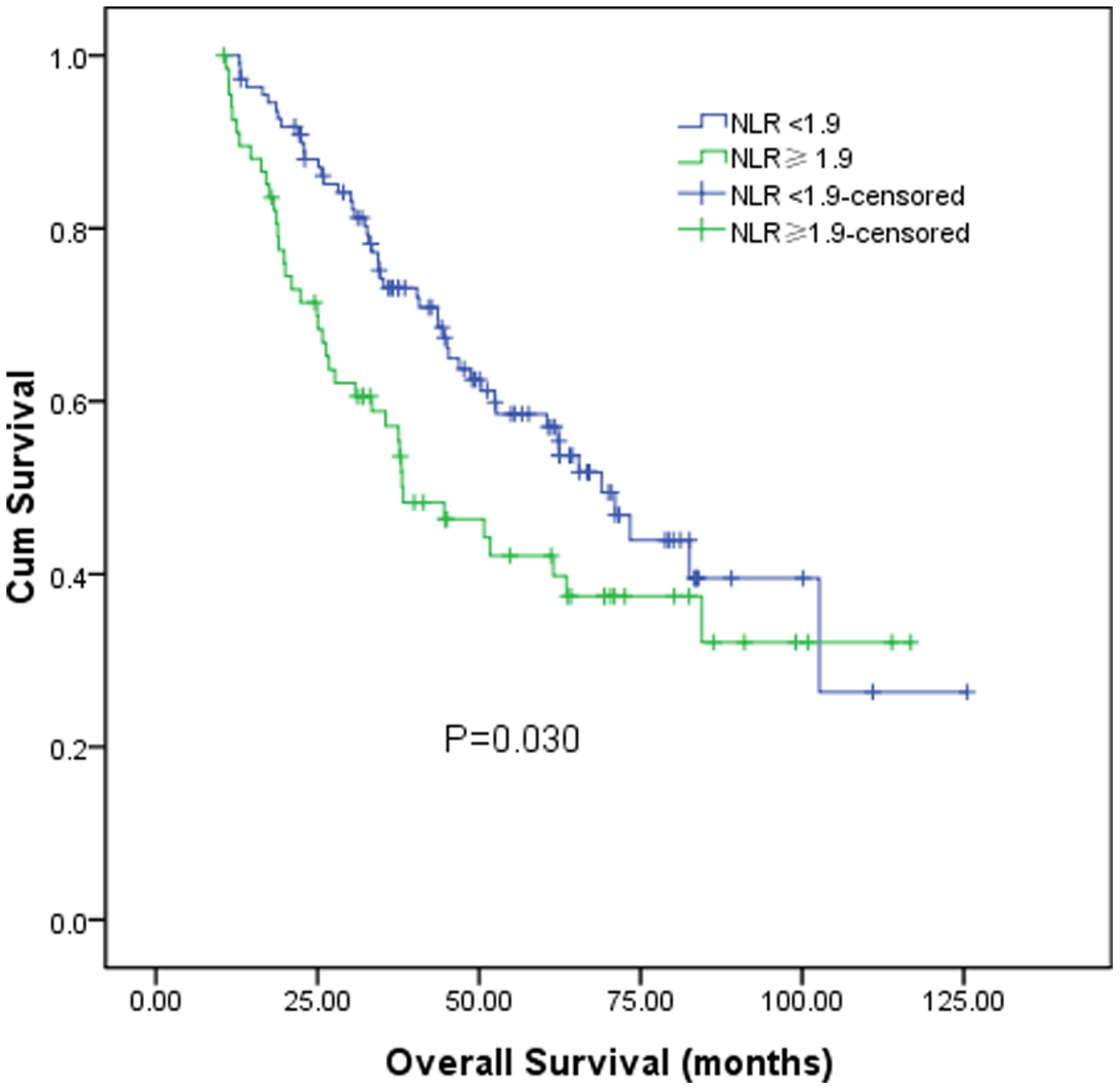
Graphs show the overall survival curves for patients with preoperative NLR≥1.9 and NLR<1.9. The difference between groups were statistically significant (log-rank test, *P* = 0.030).

After a median follow-up of 52.7 months (range 5.4–125.5 months), 148 patients (83.1%) developed tumor recurrence, and 88 patients (49.4%) died. The 1, 3, 5 years overall survival for these 178 patients was 95.5%, 67.8%, and 51.3% respectively. According to the preoperative NLR level, the 1, 3, 5 years overall survival for patients with preoperative NLR≥1.9 was 89.6%, 57.1%, and 42.1% respectively, and 99.1%, 73.1%, and 57.1% respectively for patients with preoperative NLR<1.9 (log-rank test, *P* = 0.030, Figure 1).

**Figure 2:**
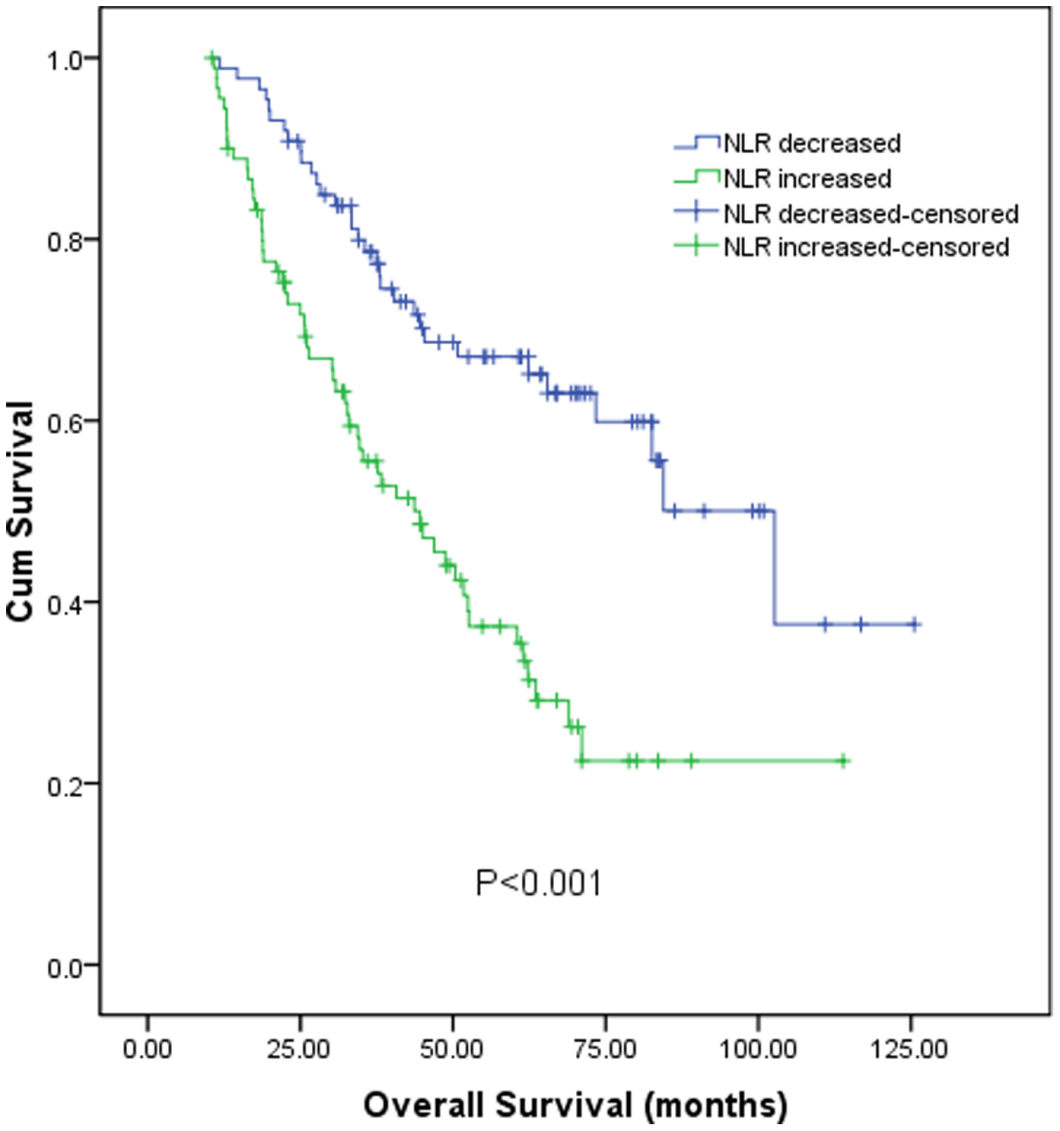
Graphs show the overall survival curves for patients with postoperative NLR decreased and increased after RFA. The difference between groups were statistically significant (log-rank test, *P*<0.001).

**Figure 3:**
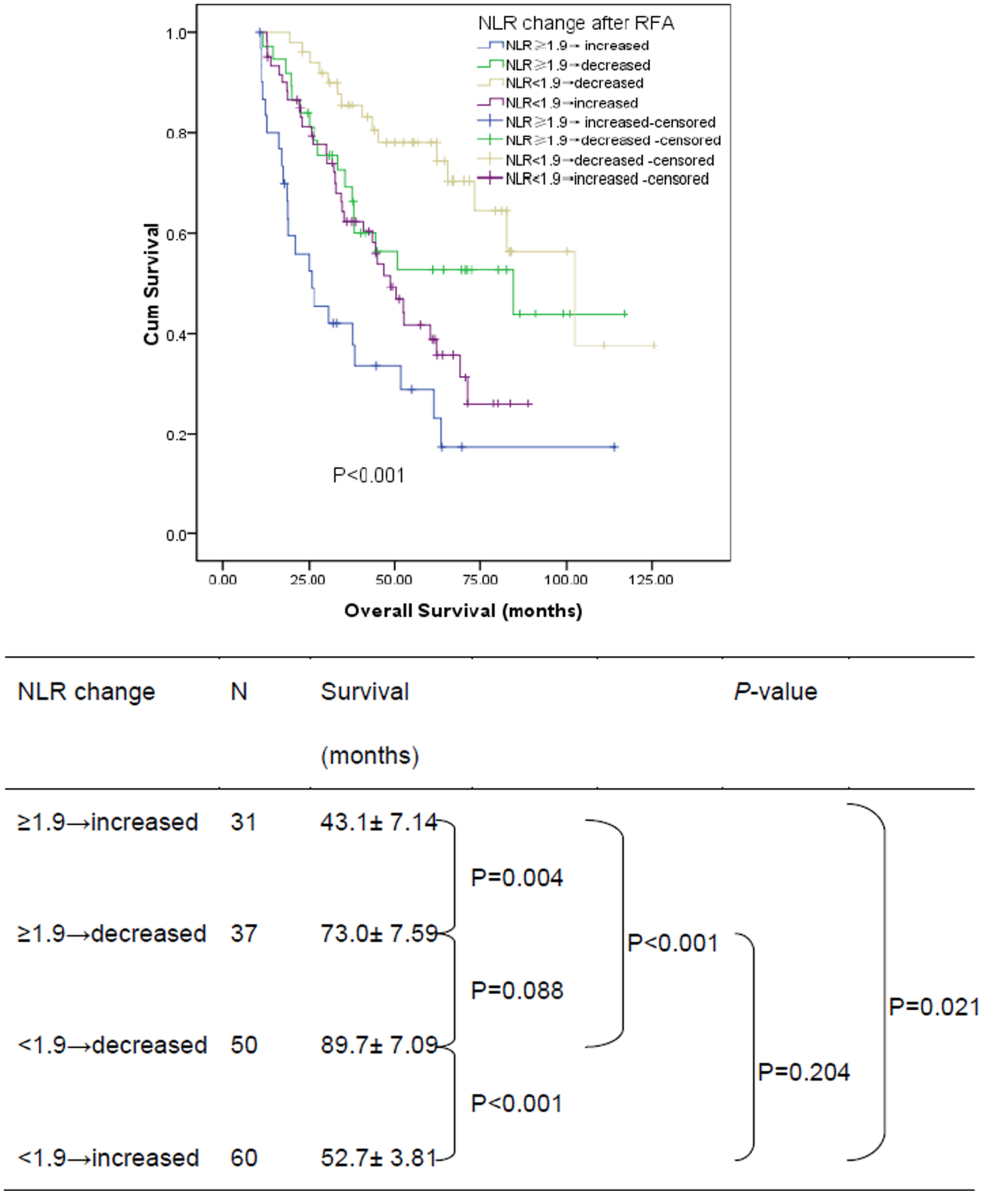
Graphs show the overall survival curves for different subgroup patients with postoperative NLR decreased and increased after RFA.

According to the postoperative NLR change, the 1, 3, 5 years overall survival for patients with decreased NLR was 98.8%, 78.6%, and 67.1% respectively, and 92.2%, 55.5%, and 35.4% respectively for patients with increased NLR. The survival of patients with increased NLR was significantly poorer than that of patients with decreased NLR (log-rank test, *P*<0.001, Figure 2). In subgroup analysis, for 110 patients with preoperative NLR<1.9, the 1, 3, 5 years overall survival for 50 patients with postoperative NLR decreased was 100.0%, 85.4%, and 77.8% respectively, and 98.3%, 62.4%, and 38.8% respectively for 60 patients with postoperative NLR increased (log-rank test, *P*<0.001, Figure 3). Similarly, for 68 patients with preoperative NLR≥1.9, the 1, 3, 5 years overall survival for 37 patients with postoperative NLR decreased was 97.3%, 69.4%, and 52.7% respectively, and 80.0%, 41.9%, and 28.7% respectively for 31 patients with postoperative NLR increased (log-rank test, *P* = 0.004, Figure 3).

As it was shown in Table 2, univariate analysis revealed that overall survival was directly influenced by tumor size, tumor number, preoperative NLR, postoperative NLR change and safety margin. Multivariate analysis showed that tumor size (*P*<0.001, HR = 2.68; 95% CI 1.72–4.17), tumor number (*P*<0.001, HR = 5.52; 95% CI 3.41–8.93) and postoperative NLR change (*P*<0.001, HR = 2.39; 95% CI 1.53–3.72) were significant prognostic factors for overall survival after RFA.

**Table 2:** Cox proportional hazards model of baseline prognosticators for overall survival in 178 patients with small HCC undergoing RFA.

		Univariate			Multivariate	
Characteristics	HR	95% CI	P-value	HR	95% CI	P-value
Age(≥65 y vs. <65y)	0.88	0.56–1.39	0.594			
Gender (Female vs. Male)	1.14	0.60–2.15	0.693			
AFP(≥400 ng/ml vs. <400 ng/ml)	1.10	0.64–1.90	0.722			
HBeAg Positive (Yes vs. No)	0.71	0.40–1.29	0.264			
Child-Pugh class(B vs. A)	1.51	0.90–2.55	0.118			
Tumor size (<3 cm vs. ≥3 cm)	2.05	1.35–3.12	0.001	2.68	1.72–4.17	<0.001
Tumor number (multiple vs. solitary)	4.47	2.85–7.00	<0.001	5.52	3.41–8.93	<0.001
Preoperative NLR (≥1.9 vs.<1.9)	1.59	1.04–2.42	0.032			
Post-NLR (decreased vs. increased )	2.53	1.63–3.93	<0.001	2.39	1.53–3.72	<0.001
Safety margin(≥0.5 cm vs. <0.5 cm)	0.60	0.40–0.91	0.017			

AFP, a-fetoprotein; CI, confidence interval; HBeAg, hepatitis B e-antigen; HR, hazard ratio; NLR, neutrophil-lymphocyte ratio; RFA, radiofrequency ablation.

### Impacts of NLR on Recurrence Free Survival

**Figure 4:**
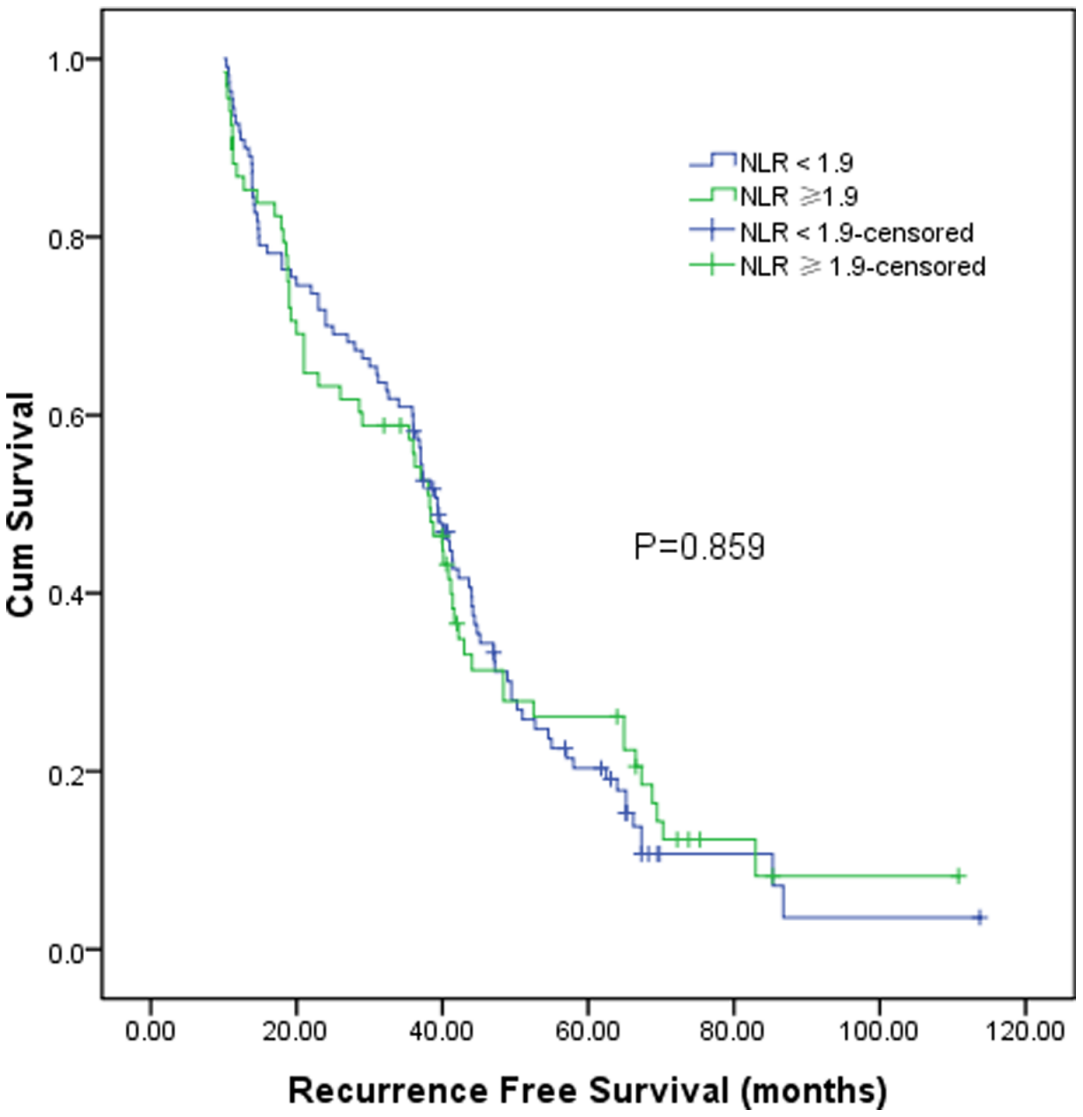
Graphs show the recurrence free survival curves for patients with preoperative NLR≥1.9 and NLR<1.9. The difference between groups were not statistically significant (log-rank test, *P* = 0.859).

The 1, 3, 5 years recurrence free survival of these 178 patients was 88.1%, 56.3%, and 23.4% respectively. According to the preoperative NLR level, the 1, 3, 5 years recurrence free survival for patients with preoperative NLR≥1.9 was 85.3%, 54.2%, and 26.1% respectively, and 90.0%, 54.2%, and 26.1% respectively for patients with preoperative NLR<1.9 (log-rank test, *P* = 0.859, Figure 4).

**Figure 5:**
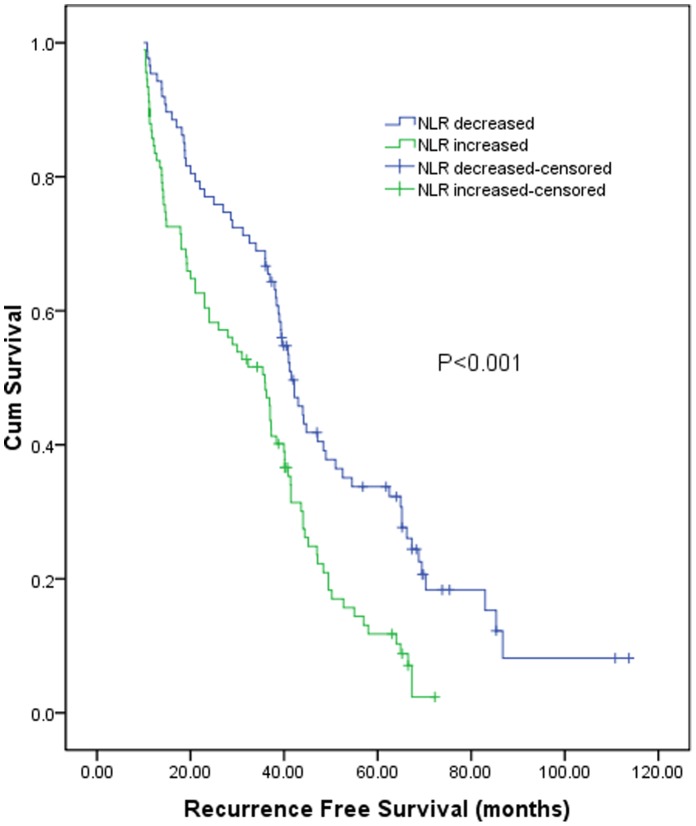
Graphs show the recurrence free survival curves for patients with postoperative NLR decreased and increased after RFA. The difference between groups were statistically significant (log-rank test, *P*<0.001).

**Figure 6:**
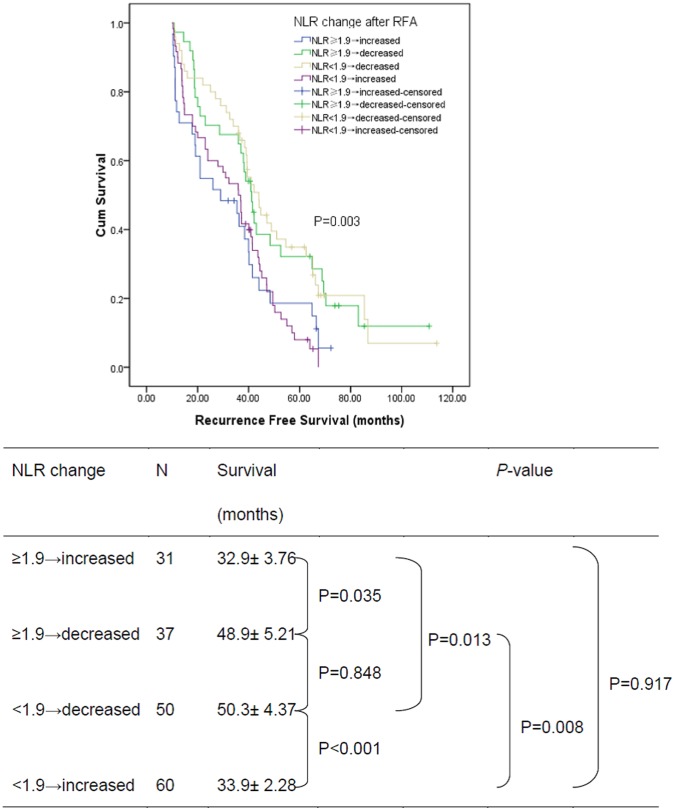
Graphs show the recurrence free survival curves for different subgroup patients with postoperative NLR decreased and increased after RFA.

According to the postoperative NLR change, the 1, 3, 5 years recurrence free survival for patients with decreased NLR was 94.2%, 65.2%, and 33.8% respectively, and 81.7%, 46.1%, and 12.4% respectively for patients with increased NLR. The recurrence free survival of patients with increased NLR was significantly poorer than that of patients with decreased NLR (log-rank test, *P*<0.001, Figure 5). In subgroup analysis, for 110 patients with preoperative NLR<1.9, the 1, 3, 5 years overall survival for 50 patients with postoperative NLR decreased was 92.0%, 65.9%, and 34.9% respectively, and 88.3%, 48.3%, and 7.9% respectively for 60 patients with postoperative NLR increased (log-rank test, *P*<0.001, Figure 6). Similarly, for 68 patients with preoperative NLR≥1.9, the 1, 3, 5 years overall survival for 37 patients with postoperative NLR decreased was 97.3%, 65.0%, and 32.3% respectively, and 71.0%, 40.9%, and 18.6% respectively for 31 patients with postoperative NLR increased (log-rank test, *P* = 0.035, Figure 6).

As it was shown in Table 3, univariate analysis revealed that recurrence free survival was directly influenced by tumor number and postoperative NLR change. Multivariate analysis showed that tumor number (*P* = 0.001, HR = 1.77; 95% CI 1.25–2.51) and postoperative NLR change (*P* = 0.003, HR = 1.69; 95% CI 1.20–2.38) were significant prognostic factors for recurrence free survival after RFA.

**Table 3:** Cox proportional hazards model of baseline prognosticators for new recurrence in 178 patients with small HCC undergoing RFA.

		Univariate			Multivariate	
Characteristics	HR	95% CI	P-value	HR	95% CI	P-value
Age(≥65 y vs. <65y)	0.97	0.68–1.37	0.849			
Gender (Female vs. Male)	1.12	0.66–1.88	0.681			
AFP(≥400 ng/ml vs. <400 ng/ml)	1.01	0.67–1.53	0.948			
HBeAg Positive (Yes vs. No)	0.80	0.49–1.30	0.369			
Child-Pugh class(B vs. A)	1.27	0.83–1.93	0.271			
Tumor size (<3 cm vs. ≥3 cm)	1.27	0.91–1.76	0.155			
Tumor number (multiple vs. solitary)	1.98	1.41–2.80	<0.001	1.77	1.25–2.51	0.001
Preoperative NLR (≥1.9 vs.<1.9)	0.97	0.69–1.36	0.860			
Post-NLR (decreased vs. increased )	1.88	1.34–2.62	<0.001	1.69	1.20–2.38	0.003
Safety margin(≥0.5 cm vs. <0.5 cm)	0.75	0.54–1.03	0.078			

AFP, a-fetoprotein; CI, confidence interval; HBeAg, hepatitis B e-antigen; HR, hazard ratio; NLR, neutrophil-lymphocyte ratio; RFA, radiofrequency ablation.

## Discussion

Previous studies suggested that systemic inflammatory markers such as NLR are useful prognostic markers for various types of cancers [22], [25], [26], [27]. However most studies focused on the pretreatment values of inflammatory markers, the dynamic change of NLR, which may reflect the dynamic change of balance between host inflammatory response and immune response, is rarely studied. In present study, for the first time, we demonstrated that postoperative NLR change was an independent prognostic factor for patients with small HCC undergoing RFA.

An elevated NLR was recognized as an independent poor prognostic factor for various malignancies [22], [25], [26], [28], [29]. In the “NLR”, “N” represents the number of circulating neutrophils, and it could represent the levels of circulating angiogenesis-regulating chemokines, growth factors and proteases which are major contributors to tumor related angiogenesis [30], [31], [32]. On the other hand, “L” represents the number of circulating lymphocyte, which have pivotal roles in cytotoxic cell death and cytokines production that inhibit proliferation and metastatic activity of tumor cells [33]. Therefore, when taken together, NLR could act as a marker that reflect the balance between host inflammatory response and immune response, and patients with elevated preoperative NLR have a relative lymphocytopenia and neutrophilic leukocytosis, which denote that the balance is tipped in favor of pro-tumor inflammatory response, and is associated with poor oncologic outcome. These findings also have been replicated in present study. Patients with higher preoperative NLR had worse prognoses than those with lower preoperative NLR.

Recently, Ohno, Y *et al*. reported that postoperative NLR change was a significant prognostic factor for tumor recurrence for patients with clear cell renal cell carcinoma after surgery [34]. It was hypothesized that postoperative NLR change might reflect the dynamic change between host inflammatory response and immune response. If NLR increased after treatment, it indicated that the balance was tipped in favor of pro-tumor inflammatory response. Otherwise, if NLR decreased after treatment, it indicated that the balance was tipped in favor of anti-tumor immune response. Our results proved this hypothesis by demonstrated that the survival of patients with increased NLR was significantly poorer than that of patients with decreased NLR. More importantly, the strength of prediction seems to be greater in postoperative NLR change than that of preoperative NLR. In current study, the preoperative NLR was not a predictor of recurrence free survival but only of overall survival, whereas the postoperative NLR change was a predictor of both recurrence free survival and overall survival. Furthermore, the survival of patients with lower or higher preoperative NLR can be distinguished more accurate by postoperative NLR change, which can also reflect the efficacy of treatment.

Postoperative NLR was also revealed to be a useful prognostic marker. Recently Chen *et al*. [35] reported that high baseline NLR was associated with worse overall survival for patients with early stage HCC after RFA; post-RFA NLR predicted not only worse overall survival, but also tumor recurrence. In their study, post-RFA NLR was hypothesized to reflect the residual host inflammation activity after tumor ablation. The high post-RFA NLR emerged as an inflammatory surrogate that host milieu was in favor of tumor recurrence/growth after tumor ablation. However, their study just focused on the postoperative NLR, but not the matched pairs of NLR from preoperative to postoperative. On the contrary, postoperative NLR change as a variable defined as postoperative/preoperative NLR in our present study, could more precisely reflect the dynamic change between host inflammatory response and immune response from preoperative to postoperative. Clinically, these NLR values can be used differently in practice. The preoperative NLR can be useful in stratifying patients before treatment, whereas the postoperative NLR change can be used for early evaluation of treatment efficiency and prediction of survival.

Theoretically, NLR was expected to decrease after RFA, because of the immune response induced by the necrosis tumor ablated after RFA. Previous reports [36], [37] described spontaneous distant tumor regression after thermal ablation, indicating a possible involvement of the immune system, hence an induction of anti-tumor immunity after thermo-induced therapy. Fagnoni F et al. [38] also reported that tumor ablation by RFA induced effects important for boosting anti-tumor immune responses. Tumor cell necrosis can generate a permanent immunogenic source of tumor antigens, and these antigens can be up-taken, processed and presented by dendritic cells for effective immunization without requirement for ex vivo antigen loading. den Brok MH et al. [39] also revealed that in situ tumor destruction can provide a useful antigen source for the induction of antitumor immunity. Induced immune response after RFA is mostly weak, and is not sufficient for the complete eradication of established tumors or durable prevention of disease progression, but it could produce a favorable effect on the outcome of survival, which could account for why patients with NLR decreased after RFA had better outcomes. But in some conditions, the host inflammatory response is in favor of tumor recurrence/growth and weigh over the induced anti-tumor immune response. The balance is tipped in favor of pro-tumor inflammatory response; the postoperative NLR remains high and predicts poor oncologic outcomes. Interestingly, for 9 patients with residual viable tumor after the first RFA, NLR was decreased in 4 patients and increased in 5 patients, and when the tumor was completely ablated after the second RFA, the NLR change was not changed in these 9 patients, namely NLR was decreased in the same 4 patients and increased in the same 5 patients. It may indicate that the balance between host inflammatory response and immune response did not change after the first and second RFA, but the underlying mechanisms need to be studied further in the future.

There are some limitations in this study. Firstly, this is a retrospective analysis from one single institution, and the case number is relatively small. Secondly, we did not evaluate postoperative CRP change together with NLR, because it is not routinely measured in our daily practice. Thirdly, the host’s immune condition may be different in cirrhosis-associated HCC compared with HCC not associated with cirrhosis. Although no previous studies reported the correlation between NLR profile and cirrhosis, there might be some heterogeneity within this study because we included HCC patients with cirrhosis and patients without cirrhosis.

In conclusion, it was firstly demonstrated in current study that postoperative NLR change was an independent prognostic factor for patient with small HCC undergoing RFA, and patients with decreased NLR predicted better survival than those with increased NLR.

## Materials and Methods

### Ethics Statement

The research was approved by the institutional review board (IRB) of Sun Yat-sen University Cancer Center, and written informed consent was obtained from each patient involved in the study.

### Patients

This is a retrospective study based on prospectively collected data. Between May 2005 and Aug 2008, patients who were newly diagnosed HCC and received RFA as initial treatment in Department of Hepatobiliary Surgery, Sun Yat-Sen University Cancer Center (Guangzhou, China) were retrospectively recruited in this study. Written informed consent was obtained before treatment. The diagnosis was made when two imaging techniques showing typical features of HCC or positive findings on one imaging study together with an alpha fetoprotein (AFP) level of >400 ng/mL, or if there was a cytological/histological diagnosis of HCC [40]. All patients were tested for hepatitis B surface antigen (HBsAg), antibody to HBsAg (anti-HBs), hepatitis B e antigen (HBeAg), antibody to HBeAg (anti-HBe), antibody to hepatitis core antigen (anti-HBc), antibody to hepatitis C virus (anti-HCV), HBV DNA, serum alanine aminotransferase (ALT), aspartate aminotransferase (AST), creatinine (Cr), blood urea nitrogen (BUN), albumin, bilirubin, alkaline phosphatase, γ-glutamyltransferase (GGT), prothrombin time (PT), activated partial thromboplastin time (APTT), alpha-fetoprotein (AFP), white blood cell count (WBC) and platelet count (PLT) before treatment.

In this study, inclusion criteria were: 1) small HCC (solitary tumor ≤5 cm in diameter or ≤3 nodules ≤3 cm in diameter); 2) no extrahepatic disease; 3) lesions being visible on ultrasound (US) and with an acceptable/safe path between the lesion and the skin as shown on US; 4) adequate baseline liver function (Child–Pugh grade A or B) and renal function (serum creatinine <124 mmol/L), and 5) no clinical symptom or signs of sepsis.

Patients with any of the following were excluded: loss to follow up within 3 months after treatment; obstructive jaundice; hepatic encephalopathy; Child–Pugh C; any other malignancy; concurrent non-malignant severe illness; patients with coexistent hematologic disorders, or known active infection at the time of blood sampling for NLR and patients with poor data integrity.

### Calculation and Definition of NLR Change

All preoperative white cells and differential counts were taken within 3 days before RFA procedure, and none of the patients had clinical symptom or signs of sepsis or received any preoperative treatments. The NLR was calculated from the differential count by dividing the absolute neutrophil count by absolute lymphocyte count. Post-operative NLR was obtained at the first follow-up visit (one month after RFA). If postoperative/preoperative NLR>1, then postoperative NLR change was defined as increased, otherwise it was defined as decreased.

### Radiofrequency Ablation Procedure

Percutaneous radiofrequency ablation procedure had been described previously [41]. In brief, RFA was performed under real-time ultrasound (EUB-2000, HITACHI Medical Systems) guidance. We used a commercially available system (RF 2000; Radio Therapeutics, Mountain View, California, USA) and a needle electrode with a 15-gauge insulated cannula with 10 hook-shape expandable electrode tines with a diameter of 3.5 cm at expansion (LeVeen; RadioTherapeutics, Mountain View, California, USA). Patients were placed in the supine position. Local anesthetic with 1% lidocaine was injected from the insertion site in the skin down to the peritoneum along the planned puncture track. The skin was incised with a small lancet, and a needle was advanced to the chosen area. Conscious analgesic sedation by intravenous fentanyl citrate and droperidol was applied before the procedure. And the patients were closely monitored during the procedure. During RFA, a hyperechoic area was observed around the electrode tip on ultrasonic monitoring. The aim of the treatment was to have this hyperechoic area covering an area larger than 1 cm around the HCC. At the end of the procedure, the generator was reactivated to ablate the needle tract to prevent bleeding.

### Follow-up

AFP, liver function test, white cells and differential counts, and dual-phase spiral computed tomography (CT) was performed one month after treatment. Assessment of response was based on the modified European Association for the Study of the Liver (EASL) criteria. Residual viable tumor tissue was considered to be present if enhancement areas were seen within the tumor at either the arterial or the portal venous phase. Additional treatment with RFA was given.

Follow-up of patients included physical examination, AFP, liver function test, white cells and differential counts, and abdominal computed tomography (CT) every 3 months in the first 2 years, and every 6 months thereafter. Chest radiography was done every 6 months to observe lung metastasis. If necessary, CT of the chest, bone scintigraphy, positron emission tomography (PET), and biopsy were also performed for the diagnosis of metastasis and/or recurrence. The last follow-up date was October 2012.

Causes of death and sites of recurrence were determined from death certificates, medical interviews, and radiological findings. Overall survival time was defined as the interval between the RFA treatment and death or the last follow up. Recurrence free survival time was defined as the interval between the RFA treatment and the first time of detectable recurrence. The treatments for recurrent tumours were determined by our multidisciplinary team (MDT) including surgeons, oncologists, radiologists, hepatologists, and pathologists.

### Statistical Analysis

The statistical analyses were performed using the SPSS 13.0 statistical software (SPSS Company, Chicago, Illinois, USA). Categorical variables were compared using the X^2^-test or Fisher’s exact test when appropriate, and continuous variables were compared using the independent sample t-test. The overall and recurrence free survivals were calculated using Kaplan-Meier method. The survival curves were constructed by Kaplan-Meier method and compared by log-rank test. The prognostic varieties in predicting overall and recurrence free survival were assessed by multivariate Cox proportional hazards regression analysis. All statistical tests were two-sided, and a significant difference was considered when *P*<0.05.
